# Island Ancestors and New World Biogeography: A Case Study from the Scorpions (Buthidae: Centruroidinae)

**DOI:** 10.1038/s41598-018-33754-8

**Published:** 2019-03-05

**Authors:** Lauren A. Esposito, Lorenzo Prendini

**Affiliations:** 1 Scorpion Systematics Research Group, Division of Invertebrate Zoology, American Museum of Natural History, Central Park West at 79th Street, New York, NY 10024-5192 USA; 2 Graduate School and University Center, City University of New York, 365 5th Avenue, New York, NY 10016 USA; 3 Essig Museum of Entomology, 130 Mulford Hall, University of California, Berkeley, CA 94720-3114 USA; 4 Present Address: Institute for Biodiversity Science and Sustainability, California Academy of Sciences, 55 Music Concourse Drive, San Francisco, CA 94118 USA

## Abstract

Scorpions are an excellent system for understanding biogeographical patterns. Most major scorpion lineages predate modern landforms, making them suitable for testing hypotheses of vicariance and dispersal. The Caribbean islands are endowed with a rich and largely endemic scorpion fauna, the origins of which have not been previously investigated with modern biogeographical methods. Three sets of hypotheses have been proposed to explain present patterns of diversity in the Caribbean: (1) connections via land bridges, (2) vicariance events, and (3) overwater dispersal from continents and among islands. The present study investigates the biogeographical diversification of the New World buthid scorpion subfamily Centruroidinae Kraus, 1955, a clade of seven genera and more than 110 species; infers the ancestral distributions of these scorpions; and tests the relative roles of vicariance and dispersal in the formation of their present distributions. A fossil-calibrated molecular phylogeny was estimated with a Bayesian criterion to infer the dates of diversification events from which ancestral distributions were reconstructed, and the relative likelihood of models of vicariance vs. dispersal, calculated. Although both the timing of diversification and the ancestral distributions were congruent with the GAARlandia land-bridge hypothesis, there was no significant difference between distance-dependent models with or without the land-bridge. *Heteroctenus* Pocock, 1893, the Caribbean-endemic sister taxon of *Centruroides* Marx, 1890 provides evidence for a Caribbean ancestor, which subsequently colonized Central America and North America, and eventually re-colonized the Greater Antilles. This ‘reverse colonization’ event of a continent from an island demonstrates the importance of islands as a potential source of biodiversity.

## Introduction

Scorpions are an excellent system for understanding biogeographical patterns. They represent one of the oldest invasions of terrestrial habitats by arthropods, having colonized land as early as 440 mya^1^. Their presence on contemporary landmasses predates many historical geological events^2^ making them good candidates for testing models of vicariance and dispersal. Additionally, different lineages of scorpions have different dispersal abilities. Some are narrowly endemic, with stenotopic habitat requirements and low vagility^3^. Others are widespread and opportunistic, with eurytopic habitat requirements, making them good dispersers. Scorpions have sufficient representation in the fossil record^2^ allowing for the inference of fossil-calibrated molecular phylogenies that could be used to calculate divergence times and test competing hypotheses concerning historical biogeography^4,5^.

**Figure 1:**
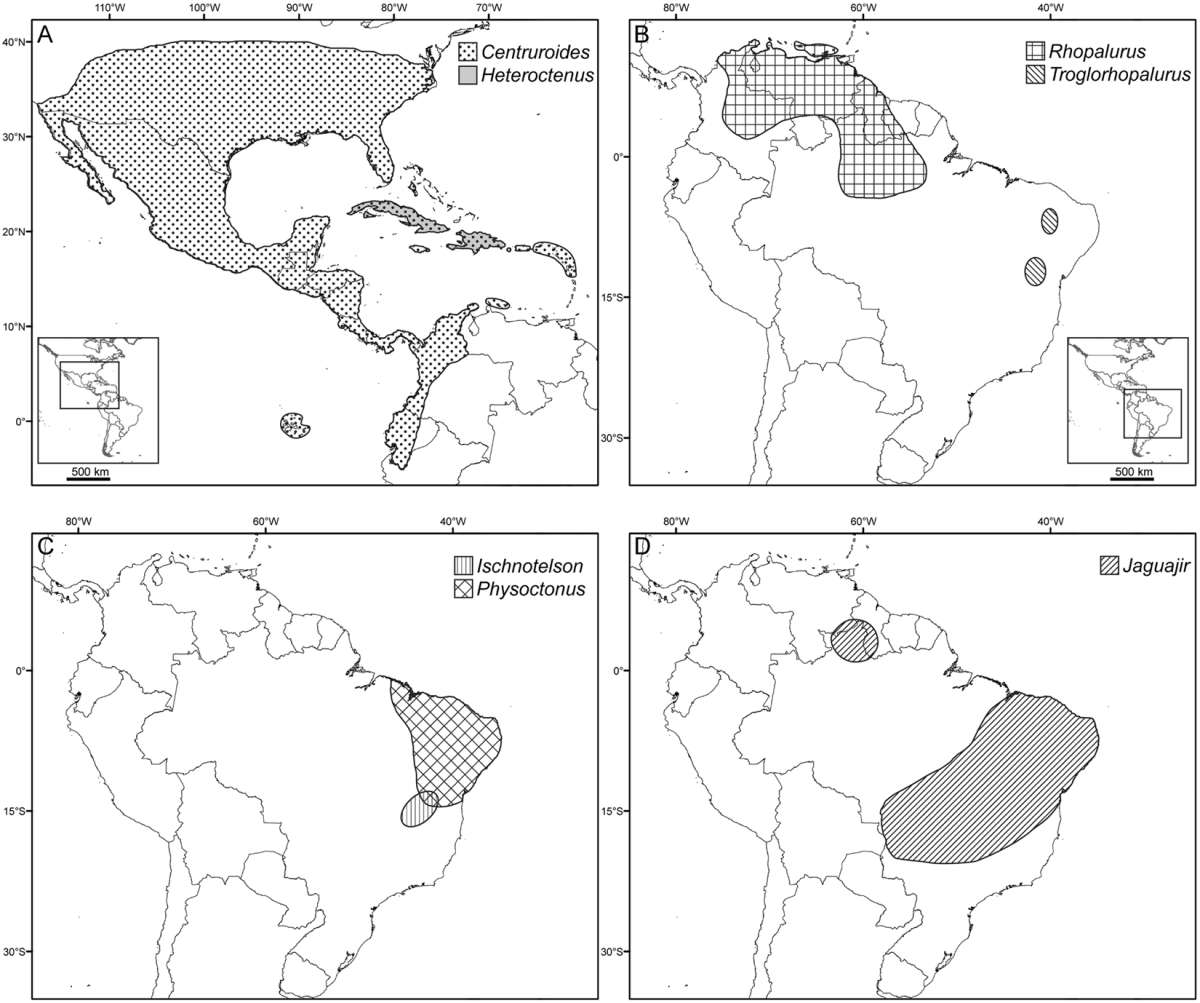
Present distributions of genera in the New World buthid scorpion subfamily Centruroidinae Kraus, 1955 [Reproduced with permission from 9]. (**A**) *Centruroides* Marx, 1890 and *Heteroctenus* Pocock, 1893. (**B**) *Rhopalurus* Thorell, 1876 and *Troglorhopalurus* Lourenço *et al*., 2004. (**C**) *Ischnotelson* Esposito *et al*., 2017 and *Physoctonus* Mello-Leitão, 1934. (**D**) *Jaguajir* Esposito *et al*., 2017.

Some of the most diverse and medically important scorpion genera occur in the New World buthid subfamily Centruroidinae Kraus, 1955. Genus *Centruroides* Marx, 1890, comprising more than 90 described species^6^, distributed from the southern United States, Mexico, Central America and the Caribbean to northern South America (Fig. 1A), is implicated in envenomations across the region. These scorpions occur on most Caribbean islands and islets (including atolls and volcanic islands) and often inhabit dead or decaying vegetation, e.g., beneath the peeling bark of trees, suggesting they are good rafters^1^. Other centruroidine genera have narrower habitat requirements and disjunct distributions, attributed to vicariance, in the savannah-grasslands of eastern and northwestern South America and the Greater Antillean islands of Cuba and Hispaniola (Fig. 1B–D)^6–8^. The paraphyly of genus *Rhopalurus* Thorell, 1876 with respect to *Centruroides* and other centruroidine genera was recently demonstrated^6,9^, resulting in a taxonomic revision, and suggesting that vicariance and dispersal played pivotal roles in generating the present diversity and distribution of centruroidine scorpion taxa inhabiting the Caribbean islands (*Centruroides* and *Heteroctenus* Pocock, 1893), North America (*Centruroides*) and South America (*Ischnotelson* Esposito *et al*., 2017, *Jaguajir* Esposito *et al*., 2017, *Physoctonus* Mello-Leitão, 1934, *Rhopalurus* and *Troglorhopalurus* Lourenço *et al*., 2004).

The biodiversity of the Caribbean islands has complex origins resulting from vicariance and dispersal events and adaptation to a diverse array of habitats. The Caribbean contains over 7000 islands distributed across an area almost 4000 km wide, and comprising ecosystems ranging from semi-desert to evergreen forest, from sea level to 3098 m elevation.

The region is comprised, principally, of three island groups: the Greater Antilles, the Lesser Antilles, and the islands of the Bahamian Bank. The Greater Antilles consist of the ‘fragment islands’ of Cuba, Jamaica, Hispaniola and Puerto Rico. Whereas Cuba and western Jamaica are probably fragments of continental origin^10^, the rest of the present-day Greater Antilles originated as the ‘proto-Greater Antilles’ when subduction of the oceanic lithosphere between the North American and South American plates created a hotspot of island formation during the Cretaceous (145–65 mya)^11–13^. As the Caribbean plate moved eastward between the continents of North and South America, the proto-Greater Antilles began to drift and fragment into smaller islands^10,14^. When it eventually collided with the shallow Bahamian Bank in the northern Caribbean Basin, the now fragmented proto-Greater Antilles entered a renewed period of subduction, vulcanism, and orogeny^13,15^. The formation of the Cayman Trench, a major fault south of Cuba, pushed western Jamaica, a fragment of the Chortis Block (present-day Guatemala, Honduras and Nicaragua^16,17^), towards the Greater Antilles. Subsequent alterations in the relative configurations of the landmasses of the Greater Antilles complicated their historical biogeography still further^18^.

The Lesser Antilles are a younger (ca. 20 mya) formation of ‘Darwinian islands’^14^ comprised of an active volcanic arc to the west and a series of islands, created by the uplift of marine sediments during movement of the Caribbean plate, to the east^18^. Excepting a few small groups of islands connected by shallow banks, there have been no direct connections among the Lesser Antilles or the continental crust^18^. The Bahamian islands are an old, stable, geologically distinct chain of ‘platform islands’ formed from the accumulation of carbonate marine sediments^19,20^.

Considering this geological context, three sets of hypotheses have been proposed to explain present patterns of diversity in the Caribbean islands: (1) connections via land bridges, (2) vicariance events, and (3) overwater dispersal from continents and among islands. Evidence from plants and herpetofauna^21–28^ has been used to argue that the absence of lineages predating the breakup of the proto-Greater Antilles precludes a vicariance origin. Based on this evidence, researchers suggested that the initial colonization of most Caribbean island taxa occurred via overwater dispersal on flotsam from neighboring continents. Other studies of mammals^10,29^, lizards^10,19,24^ and plants^27^ suggested that vicariance played a larger role than dispersal in the initial colonization of the Caribbean islands.

One vicariance hypothesis, the GAARlandia (Greater Antilles + Aves Ridge) hypothesis^10^, was proposed to explain the disjunct distribution of some mammal taxa in the Greater Antilles and South America. Using geological data, including fossil evidence, Iturralde-Vinent and MacPhee (1999) proposed the existence of a land bridge connecting South America to the Greater Antilles during the Eocene–Oligocene transition, 35–33 mya^10^. The proposed subsequent break-up of the proto-Greater Antilles emphasizes the role of vicariance in the diversification of Antillean biota. The GAARlandia hypothesis was tested using molecular evidence from several plant^30–34^, mammal^29^, and arachnid^35^ lineages, with conflicting results. Whereas the divergence times of some lineages were consistent with the model, others did not fit its predictions. Dávalos (2004), drawing from mammal data, rejected a general biogeographical pattern for the initial colonization of the Greater Antilles, and suggested that patterns should be treated on a taxon-by-taxon basis^29^.

The origins and affinities of the rich and largely endemic scorpion fauna of the Caribbean islands have not been previously investigated with modern biogeographical methods. The present study provides the first fossil-calibrated phylogeny for the New World buthid scorpion subfamily Centruroidinae, which is used to infer the relative roles of vicariance and dispersal in the formation of their present distributions. Hypotheses of vicariance vs. dispersal are tested with and without the GAARlandia land-bridge, the distributions of ancestral nodes inferred, and diversification dates estimated at nodes of interest.

## Results

### Phylogeny

**Figure 2:**
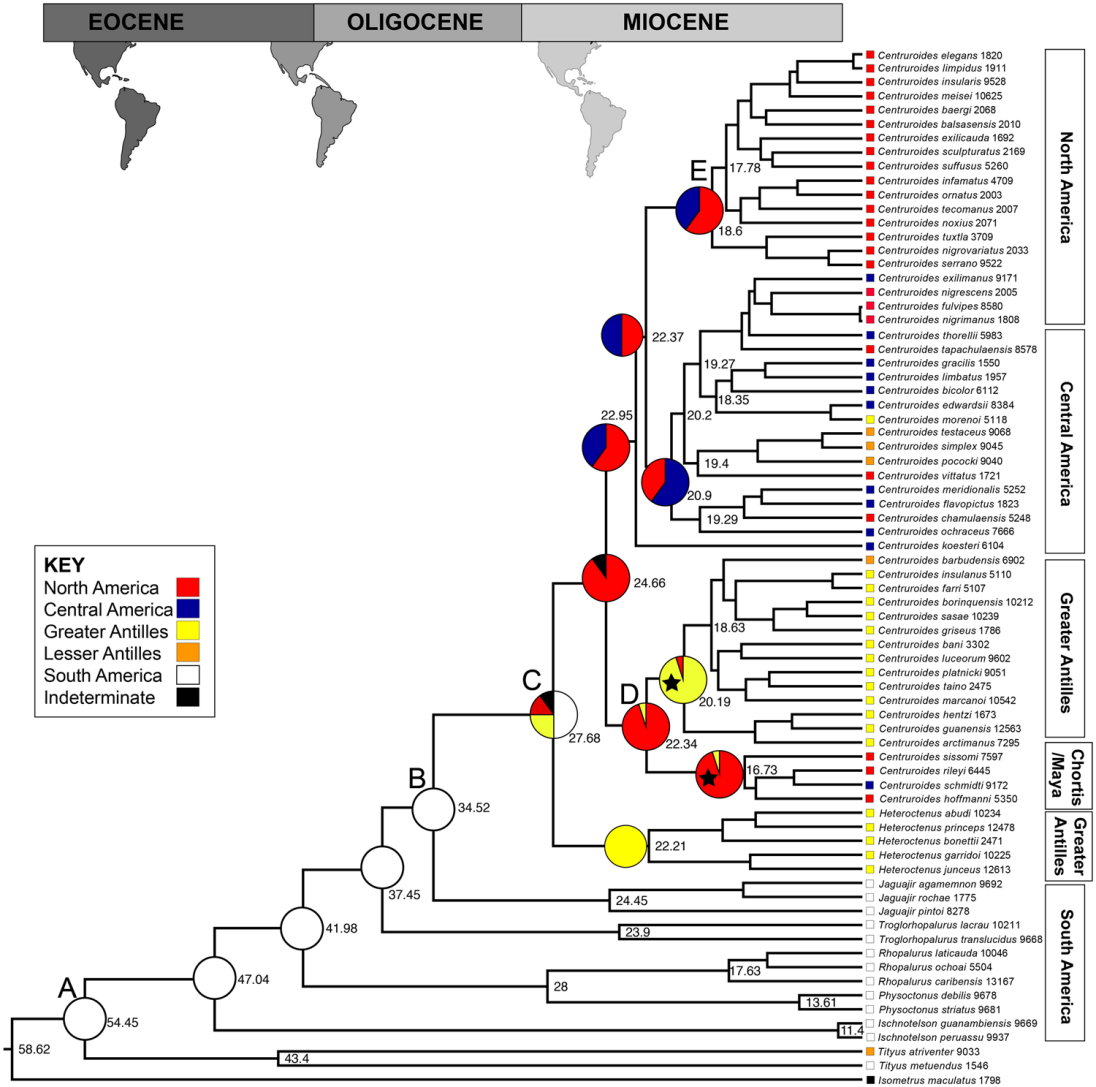
Fossil-calibrated phylogram of the New World buthid scorpion subfamily Centruroidinae Kraus, 1955. Maximum Clade Credibility phylogram inferred in BEAST^36^ and congruent with results from Maximum Likelihood inference in RAxML^37^. Labels to right of nodes indicate median ages calculated in BEAST. Pie charts to left of nodes indicate ancestral distributions calculated using a Bayesian criterion in RASP^38^. Calibration points indicated with stars. Map reconstructions at top reflect hypothesized landmasses^10,48^ available during corresponding time periods. Squares to left of taxon names indicate present distributions (see legend). Inset photos to the left are representative of the major clades of *Centruroides*: 1) *Centruroides scultpuratus* (Ewing, 1928), 2) *Centruroides hentzi* (Banks, 1900), 3) *Centruroides gracilis* Latreille, 1904, 4) *Centruroides rileyi* Sissom, 1995.

The tree topologies recovered by two independent RAxML^29^ runs were congruent, indicating that the tree with the highest likelihood (−47574.55) was successfully recovered (Fig. 2, S1). All centruroidine genera were recovered as monophyletic with high bootstrap support (BS = 100). As in previous analyses^6,9^, *Centruroides* and *Heteroctenus* formed a well-supported sister group (BS = 99). Four distinct clades of *Centruroides* were recovered with high support, i.e., a North American clade (BS = 100), a Central American clade (BS = 94), a Greater Antilles clade (BS = 90), and a clade representing the Chortis and Mayan Blocks (BS = 99), where the Chortis Block is defined as the part of Central America south of the Motagua Fault in Guatemala and north of latitude 12°30’N and the Mayan Block is defined as the area north of the Motagua fault and southwest of the Salina Cruz fault that crosses the Isthmus of Tehuantepec^16,17^.

### Divergence Time Estimation

The topology of the fossil-calibrated phylogeny estimated with BEAST^36^ (Fig. 2, S1) was identical to the topologies recovered with RAxML^37^. Effective sample size for all parameters was >300. Node A depicts the outgroup-constrained split between *I. maculatus* and the New World buthid exemplars, with a 95% confidence interval of {48, 66} mya. Node B depicts the split between the clade comprising *Centruroides* and *Heteroctenus*, and the remaining centruroidine genera, with a 95% confidence interval of {31, 38} mya. Node C depicts the split between *Centruroides* and *Heteroctenus* with a 95% confidence interval of {26, 30} mya. Node D depicts the split between the Greater Antilles clade and the Chortis/Maya clade of *Centruroides* with a 95% confidence interval of {23, 26} mya. Lastly, Node E depicts the North American clade of *Centruroides* with a 95% confidence interval of {22, 24} mya.

### Ancestral Distribution Reconstruction

Ancestral distributions inferred under parsimony and Bayesian criteria^38^ were largely congruent. The ancestral node of New World buthids was reconstructed as South American in both analyses. The distribution of the common ancestor of *Centruroides* and *Heteroctenus* was unresolved in the parsimony analysis but was recovered principally as (Greater Antilles + South America), with <25% recovered as North American and undetermined, in the Bayesian analysis. The ancestral distribution of *Centruroides* was also unresolved in the parsimony analysis but was recovered as North American in the Bayesian analysis. All ancestral clade distributions within *Centruroides* were unresolved in the parsimony analysis, except for the Chortis/Maya clade and the Greater Antilles clade, both of which were recovered as North American. The Bayesian analysis also recovered a North American ancestral distribution for the Chortis/Maya clade and the Greater Antilles clade, in addition to recovering an ancestral (North America + Central America) distribution for the North American and Central American clades.

### Biogeographical Hypothesis Testing

The biogeographical hypothesis testing in Lagrange^39,40^ identified no significant differences between any pair of hypotheses (Table 1). The likelihood values for dispersal-only models and the dispersal + vicariance (GAARlandia) models were equally probable. A significant difference in the likelihood values was observed among models with varying dispersal influence, however. The likelihood scores of models with dispersal were significantly higher (less probable) than those of models without dispersal, and the scores of models in which dispersal was dependent on distance were lower (more probable) than those of models in which dispersal was independent of distance.

**Table 1:** Lagrange^39,40^ analyses of the relative importance of dispersal and vicariance, associated with the GAARlandia land bridge^10^ in forming the present distribution of the New World buthid scorpion subfamily Centruroidinae Kraus, 1955.

Model	−ln(L)	Dispersal	Extinction
Distance-dependent dispersal without GAARlandia	−69.28	0.0036	0.002
Distance-dependent dispersal with GAARlandia	−69.18	0.0036	0.002
Dispersal without GAARlandia	−69.86	0.0039	0.002
Dispersal with GAARlandia	−69.82	0.0039	0.002
No dispersal without GAARlandia	−76.68	0.0276	0.002
No dispersal with GAARlandia	−75.32	0.0283	0.002

## Discussion

The origins and diversification of the Caribbean biota are fascinating because of the complex geological history of the Caribbean islands and the proximity of major continental landmasses (North, Central, and South America) containing dramatically different habitats. The proximity of some Caribbean islands to continents has facilitated biotic exchange between these landmasses. Historically, island biogeography and metapopulation theory^41^ viewed island–continental biotic interchange as strictly unidirectional, with larger landmasses acting as sources and islands as sinks for biodiversity^42,43^. However, increasing evidence^44^ points to a paradigm shift where islands, at least in the Caribbean, are no longer considered the end of the road^18^. ‘Reverse colonization’ of continents from islands has been demonstrated in animal taxa as diverse as amphibians, birds, insects, mammals, and reptiles^45^. As similar mechanism may explain the diversification of Caribbean centruroidine scorpions.

Starting at the base of the phylogeny from the BEAST analysis (Fig. 2, Node A), the node age is consistent with the rifting of South America from Antarctica in the Eocene (56–34 mya). Diversification of the major lineages of New World buthid scorpions likely occurred in South America during the late Eocene and early Oligocene. This date is congruent with that obtained in a fossil-calibrated phylogeny of South American buthid scorpions^46^. The first dispersal event out of South America involved an ancestor of *Centruroides* and *Heteroctenus* which, based on the inferred date, the Lagrange analysis, and the ancestral state reconstruction, dispersed into the Greater Antilles from northern South America, as no route for dispersal via Central America was available at the time. This pattern of distribution is consistent with the GAARlandia hypothesis^10^, according to which a land bridge existed between South America and the proto-Greater Antilles when the Aves Arc emerged briefly 35–33 mya (although the Lagrange analysis does not favor GAARlandia over a model without the possibility of the GAARlandia land bridge).

Node B, reflecting the split between *Jaguajir* and the clade comprising *Centuroides* and *Heteroctenus*, has a median age of 35.5 ± 3 mya, consistent with the proposed timing of GAARlandia. This is further supported by the Bayesian ancestral distribution reconstruction which indicates that the ancestral node of (*Centruroides* + *Heteroctenus*) had a (Caribbean + South American) distribution, although a minor part of the inferred ancestral range also includes North America. As the islands in the Lesser Antilles did not emerge until after 12 mya, a land bridge providing a route for dispersal between the Greater Antilles and northern South America best explains the reconstructed distribution. Indeed, the likelihood scores of models that assumed dispersal-only scenarios were greater than those that assumed vicariance only. In a study on *Selenops* Latreille, 1890 spiders, where the same parameters were applied to a similar distribution, Crews and Gillespie (2010), rejected the dispersal-only hypotheses in favor of GAARlandia^35^. Teruel (2006) also invoked the GAARlandia hypothesis to explain the present distributions of Caribbean centruroidine scorpions but did not offer a biogeographical analysis to support his assertions^7^.

The split between *Centruroides* and *Heteroctenus* occurred during the early Oligocene, and the diversification of the major lineages of *Centruroides* occurred during the early Miocene. This was a period of considerable geological activity in the Greater Antilles, as the Caribbean plate moved into the present-day Caribbean Basin, towards North America, before eventually coming to rest against the Bahamian Bank^10^. Geological details of the islands during this period are complex and beyond the scope of this discussion. However, island breakups in combination with submersions, collisions, and changes in sea level provided ample opportunity for further diversification of lineages in the region^18^.

According to the Bayesian analysis, the ancestral distribution of *Centruroides* was North American. The dating analysis and ancestral distribution reconstruction further suggest that the initial diversification of *Centruroides* in North America resulted from dispersal or vicariance from the Greater Antilles, consistent with the ‘out of Cuba’^47^ hypothesis. The sister-group relationship between the Greater Antilles clade and the Chortis/Maya clade of *Centruroides* suggests a possible route for colonization of the Americas. The proximity of Cuba to the Mayan Peninsula and the Chortis Block during the eastward migration of the Caribbean Plate^10,27^ may have provided an opportunity for dispersal between these landmasses. The hypothesis that *Centruroides* colonized and diversified into northern Central America via dispersal from the Greater Antilles was suggested for eleutherodactyline frogs^27^. The complexity of Caribbean geography makes reconciling the precise routes of dispersal difficult, however. Dispersal to North America from South America, and subsequent dispersal to the Caribbean, is also plausible, based solely on the ancestral distributions, but these area reconstructions ignore the availability of land bridges for dispersal by animals with limited vagility, at the dates inferred. Explicit hypothesis-testing, considering the availability of land bridges at particular times, e.g., closure of the Central American seaway in the Pliocene^10,48,49^, favored the GAARlandia inclusive model of distance-dependent dispersal, albeit slightly.

The eurytopic, often arboreal habitat requirements of many *Centruroides* may have aided their colonization of the Caribbean, Central and North America, and could explain the absence in North and Central America of *Heteroctenus*, terrestrial savannah specialists restricted to the Greater Antilles.

**Figure 3:**
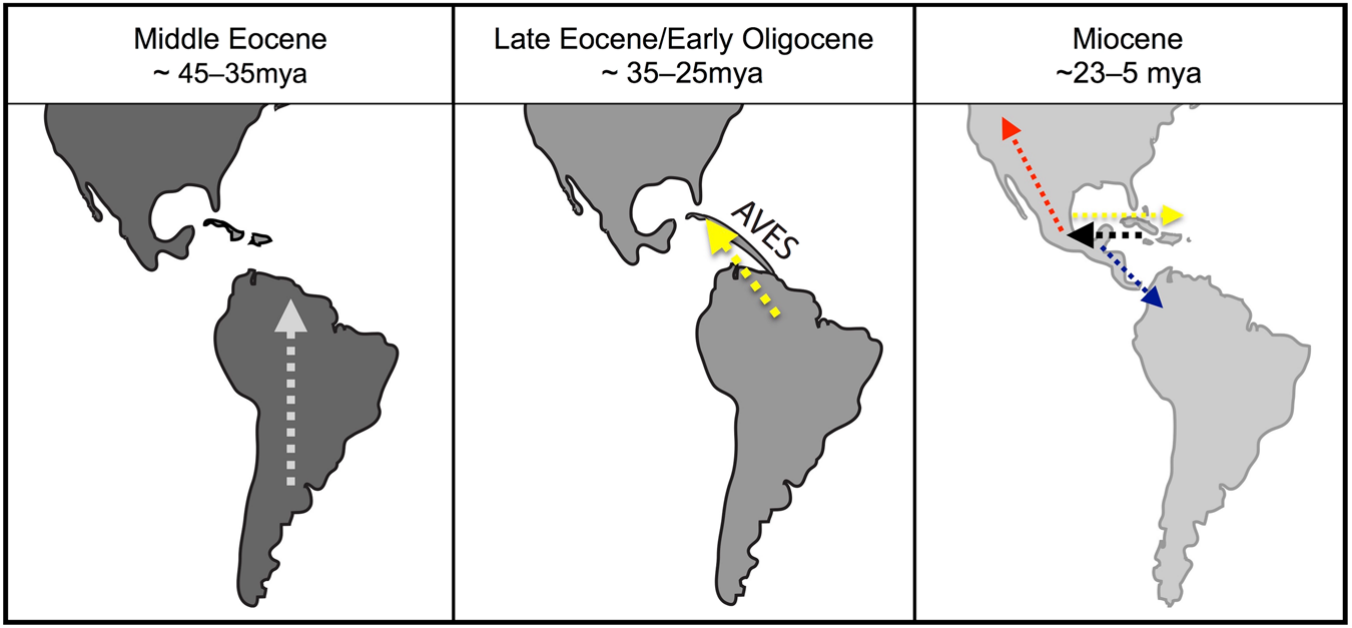
Key dispersal events in biogeographical reconstruction of the New World buthid scorpion subfamily Centruroidinae Kraus, 1955. Map reconstructions reflect hypothesized landmasses available during corresponding time periods^10,48^. Colored arrows indicate dispersal events as reconstructed in Fig. 2: dispersal of centruroidine lineages within South America during the middle Eocene; dispersal of *Heteroctonus* Pocock, 1893 from South America to the Greater Antilles during the late Eocene and early Oligocene; dispersal of major lineages of *Centruroides* Marx, 1890 during the Miocene, first from the Greater Antilles onto the continent (black arrow), then northward to North America (red arrow), southward through Central America (blue arrow), and finally eastward back to the Greater Antilles (yellow arrow).

Two major radiations of *Centruroides* appear to have occurred on the American continents: one southward, through Central America and into northern South America; the other northward, through Mexico to the southwestern United States, where *Centruroides* appears to have diversified in the newly emerging habitat of the North American deserts at the end of the last glacial maximum (Figs 2,3). The age of the clade of *Centruroides* species inhabiting the North American deserts (Fig. 2, Node E) is consistent with the emergence of these habitats ca. 15 mya^50^.

In addition to putative northward- and southward-trending dispersal, *Centruroides* apparently returned eastward, successfully re-colonizing the Caribbean during the Miocene (Fig. 3). The plausibility of a Caribbean re-colonization by *Centruroides* is supported by the basal position of the Cuban species of *Centruroides* within the Greater Antilles clade.

In summary, according to the reconstruction presented here, centruroidine scorpions colonized the Caribbean islands on two independent occasions (Fig. 3). The first dispersal event occurred approximately 35 mya, probably via the GAARlandia land-bridge between northern South America and the Greater Antilles. The second occurred approximately 20 mya from North America, probably via Cuba and ultimately eastward to other islands in the Caribbean archipelago. The probability of dispersal into a new geographical area appears to have been strongly dependent on the proximity of the new area to an area with an established source population. Importantly, at least one ‘reverse colonization’ event transpired, where an island ancestor dispersed onto a continent and subsequently radiated in the new environment. This demonstrates the importance of islands as a potential source for creating and/or maintaining continental biodiversity.

## Methods

### Taxon Sampling

The analysis includes 74 terminal taxa, including 71 ingroup taxa of subfamily Centruroidinae (approximately 63% of the total species diversity of the subfamily): 54 of the 94 described species of *Centruroides* (57%), five of the six described species of *Heteroctenus* (83%), and all described species of the other five centruroidine genera, *Ischnotelson*, *Jaguajir*, *Physoctonus*, *Rhopalurus*, and *Troglorhopalurus* (Appendix 1).

Based on data presented here, three subspecies of *Centruroides* are elevated to full species: *C. insularis* Pocock, 1902, stat. nov.; *C. meridionalis* Hoffmann, 1932, stat. nov.; *C. taino* Armas & Marcano Fondeur, 1987, stat. nov. Additionally, *Centruroides borinquensis* Armas, 1982, stat. rev. is removed from synonymy with *Centruroides griseus* (C.L. Koch, 1844).

Two exemplar species of another New World buthid genus, *Tityus* C.L. Koch, 1836, were included as outgroups^51,52^ and the analysis was rooted on the cosmotropical buthid, *Isometrus maculatus* (DeGeer, 1778)^9^.

### Field Methods

Personally-collected material was located at night using ultraviolet light detection, immersed in and subsequently injected with 95% ethanol, and stored at 5 °C until returning to the lab. Tissue samples (stored at −20 °C) and voucher specimens are deposited at the American Museum of Natural History, New York.

### Laboratory Methods

Genomic DNA was extracted from muscle tissue of the fourth leg of each specimen using a Qiagen DNEasy Blood and Tissue extraction kit according to the manufacturer’s protocols. Extracted DNA was amplified for five gene loci, selected based on their ability to provide resolution at various taxonomic levels^53–58^, in overlapping fragments using universal eukaryote and scorpion specific primers (Table 2): a mitochondrial protein-coding gene, Cytochrome *c* Oxidase subunit I (COI), two mitochondrial structural genes, 12S rDNA (12S) and 16S rDNA (16S), and two nuclear structural genes, 18S rDNA (18S) and 28S rDNA (28S).

**Table 2:** Primers used for amplification of 12S rDNA (12S), 16S rDNA (16S), 18S rDNA (18S), 28S rDNA (28S) and Cytochrome *c* Oxidase subunit I (COI) in a fossil-calibrated biogeographical analysis of the New World buthid scorpion subfamily Centruroidinae Kraus, 1955.

Gene	Primer	Other names	Sequence	References
12S	12Sai	SR-N-14588	AAACTAGGATTAGATACCCTATTAT	^70^
	12Sbi	SR-J-14233	AAGAGCGACGGGCGATGTGT	^70^
16S	16Sar	LR-N-13398	CGCCTGTTTATCAAAAACAT	^71^
	16Sbr	LR-J-12887	CTCCGGTTTGAACTCAGATCA	^71^
18S	18S1F		TACCTGGTTGATCCTGCCAGTAG	^72^
	18S5R		CTTGGCAAATGCTTTCGC	^72^
	18S3F		GTTCGATTCCGGAGAGGGA	^72^
	18Sbi		GAGTCTCGTTCGTTATCGGA	^73^
	18SA2.0		ATGGTTGCAAAGCTGAAAC	^73^
	18S9R		GATCCTTCCGCAGGTTCACCTAC	^72^
28S	28Sa	D3A	GACCCGTCTTGAAGCACG	^74^
	28Sbout		CCCACAGCGCCAGTTCTGCTTACC	^54^
COI	LCO	LCO-1490-J-1514	GGTCAACAAATCATAAAGATATTGG	^75^
	HCOoutout		GTAAATATATGRTGDGCTC	^76^
	LE1R		TCCATTCCCACAGTAAACATATG	^5, 9^
	HCOEXTERNA		GAAGTTTATATTTTAATTTTACCTGG	^71^
	HCOEXTERNB		CCTATTGAWARAACATARTGAAAATG	^71^

The Polymerase Chain Reaction was performed in an Epicenter thermocycler (Eppendorf) using GoTaq polymerase (Promega). Reactions were verified on a 1.2% agarose gel stained with Sybr safe DNA gel stain (Invitrogen), and subsequently purified using the Ampure DNA (Agencourt) purification system on a Biomek NX robot (Beckman-Coulter).

Cycle sequencing was conducted using Big Dye v1.1 and automated Sanger sequencing of single-stranded DNA performed on an Applied Biosystems Inc. Prism™ 3730×. Paired-strand reads were aligned using Sequencher™ and edited by hand. A total 370 DNA sequences were generated (Appendix 1). The sequences of all exemplars were complete for all five gene loci.

### Phylogenetic Methods

Multiple sequence alignments for individual gene partitions were performed in MAFFT^59,60^ using the G-INS-i strategy, recommended for fewer than 200 sequences with global homology, and the PAM1/K = 2 matrix parameter, recommended for aligning sequences of closely related taxa. There was no length variation among the COI sequences, and trivial length variation (10–20 nucleotides) among the ribosomal DNA sequences. The resulting sequence alignments were manually checked in Geneious (Biomatters, Ltd.) and concatenated to yield a total alignment of 4250 characters, with 3104 invariant sites, 167 variable but uninformative sites, and 979 informative sites. The nucleotide composition was 25% A, 18.5% C, 25% G and 31.5% T.

The concatenated dataset, partitioned by gene and codon position, was analyzed with RAxML-HPC v7.2.7^37^. Each partition was analyzed under the GTR + Γ model^61^, as the difference between the fit of this model and other models was insignificant. RAxML employs a rapid search algorithm that quickly searches tree space but does not always recover the tree with the best likelihood, hence two runs were performed in combination with the ‘rapid bootstrap’ algorithm.

### Divergence Time Estimation

A fossil-calibrated phylogeny was estimated with BEAST v1.8.4^36^ using a relaxed molecular clock with unlinked partitions^59^. Each partition was analyzed under the GTR + Γ model. The resulting best tree from the RAxML analysis was transformed into an ultrametric tree using nonparametric rate smoothing over the branch lengths in TreeView v1.0^61^. The tree was then rooted and scaled to reflect the age and topological constraints imposed on the Bayesian priors and used as a starting tree for the BEAST analysis. An uncorrelated lognormal tree prior was used for dating, with a Yule speciation tree prior. Models and molecular clocks for each partition were unlinked. A lognormal distribution was used for fossil calibration points with the mean age of the fossil equal to the lognormal mean and the fossil dating error equal to the lognormal standard deviation.

The analysis was run for 50 million generations, sampling every 5000 generations. Burn-in times were determined by eye using ln-likelihood in Tracer v1.5^62^, and convergence assessed by the standard deviations of the split frequencies. The first 5 million generations were discarded as burn-in. A maximum clade credibility tree was computed from the post-burn-in trees using TreeAnnotator v1.8.4^36^.

### Fossil Calibration

A fossil of *Centruroides* from Dominican amber^63^ was assigned to an extant taxon by some authors^64^. Although this is unlikely given its age, the extinct species is morphologically similar to extant *Centruroides* occurring in the Dominican Republic and can be assigned to the same clade with a high degree of confidence. The precise locality of the mine from which the fossil was recovered is unknown, but all true amber occurrences in the Dominican Republic are associated with lignitic material and are of late Early to early Middle Miocene, approximately 20–17 mya^65,66^. Fossils of *Tityus*, the origins of which are also unknown, have also been described from Dominican amber^67^.

Another *Centruroides* fossil, described from amber deposits in the state of Chiapas, Mexico, is contemporaneous with Dominican amber^63,68^. The Chiapas amber specimen was only tentatively assigned to *Centruroides* and no additional assessment of its placement has been published. The morphology of the Chiapas amber specimen suggests it is related to *Centruroides hoffmanni* Armas, 1996 and related extant species occurring in Chiapas and neighboring areas^6,9^. The Chiapas amber specimen is believed to have originated from mines near Simojovel^68^. These deposits were formed from the sap of an extinct legume in the genus *Hymanaea* L.^69^. Amber-bearing deposits in the Simojovel region are part of the Mazantic shale and Balumtum sandstone strata, with a relative age estimated to be 23–15 mya^68,69^.

Minimum age constraints based on amber fossils were applied to three nodes (Table 3, indicated with stars in Fig. 2): the basal node for the outgroup exemplars of *Tityus*, based on the *Tityus* fossils present among the Dominican amber fauna; the basal node for the Greater Antilles clade of *Centruroides* to which the Dominican amber fossil is putatively assigned; and the basal node of the Chortis/Maya clade of *Centruroides* to which the Chiapas amber fossil can be tentatively assigned. The upper bound of the age range assigned to each stratum was used for the calibration (17 mya for Dominican amber, 15 mya for Chiapas amber), with a lognormal distribution and sigma equivalent to 15 mya.

**Table 3:** Calibration points and sources of dates for scorpion genera *Centruroides* Marx, 1890 and *Tityus* C.L. Koch, 1836 used in divergence dating analysis of the New World buthid scorpion subfamily Centruroidinae Kraus, 1955 in BEAST^36^.

Source	Clade	Minimum age	References
Chiapas amber	Chortis/Maya *Centruroides*	15 mya	^68, 69^
Dominican amber	Greater Antilles *Centruroides*	17 mya	^65, 66^
Dominican amber	*Tityus*	17 mya	^65, 67^

### Ancestral Distribution Reconstruction

Ancestral distributions were reconstructed under both Bayesian and parsimony criteria using RASP^38^. RASP requires three input files: a tree set (distribution of trees), a file providing the distribution of each taxon, and a consensus tree or preferred topology. The post-burn-in trees from the BEAST analysis were used for the tree set and the Maximum Clade Credibility tree was specified as the consensus tree. Taxa were assigned to one or more of six geographical regions based on their known distributions: Australasia; South America (south of the Isthmus of Panama); Central America (north of the Isthmus of Panama and south of the Isthmus of Tehuantepec); North America (north of the Isthmus of Tehuantepec); the Greater Antilles; the Lesser Antilles.

### Biogeographical Hypothesis Testing

Lagrange^39,40^ was used to test competing hypotheses of vicariance and dispersal. Lagrange uses likelihood models that consider differences in dispersal and extinction at various time periods, accounting for external information such as dispersal potential and the geological history of a region. Given the geological complexity of the Caribbean islands, it is critically important that this information be considered.

The analysis follows a study of *Selenops* spiders^35^ with a distribution resembling that of *Centruroides*, which identified six geological events essential to forming a route for dispersal between North America and South America across the Caribbean Basin (Table 4). The relative importance of vicariance and dispersal in explaining the present distribution of *Centruroides* was tested as follows: A) colonization via dispersal only (GAARlandia absent), with probability of colonization via dispersal dependent on distance; B) colonization via dispersal or vicariance (GAARlandia present), with probability of dispersal dependent on distance; C) colonization via dispersal, with probability of colonization via dispersal equal regardless of distance (GAARlandia absent); D) colonization via dispersal or vicariance (GAARlandia present), with probability of dispersal not dependent on distance (all equal); E) colonization with little dispersal (GAARlandia absent); F) colonization with little dispersal (GAARlandia present). The likelihood parameters for each hypothesis consider the Caribbean geography for time periods corresponding to the six geological events (Table 4), such that the probability of dispersal to any given landmass is dependent upon its existence at that time. For example, if the island of Grenada had not yet formed during a given time slice, the probability of dispersal to that island is zero.

**Table 4:** Geological events affecting dispersal of organisms between North and South America, following^35^.

Geological event	Age
Closure of Isthmus of Panama	3 mya
Most recent appearance of northern Lesser Antilles	5 mya
Most recent appearance of southern Lesser Antilles	12 mya
Disappearance of GAARlandia	33 mya
Appearance of GAARlandia	35 mya
Time after which land exposed in Greater Antilles	55 mya

The Lagrange analysis requires the definition of geographical regions to which distributions of taxa can be assigned. Five regions were defined for this analysis: South America, south of the Isthmus of Panama, plus Aruba, Curaçao and Bonaire; North America and Central America, north of the Isthmus of Panama; Greater Antilles, i.e., the Caribbean islands of Cuba, Hispaniola, Puerto Rico, and the Bahamian Bank; northern Lesser Antilles, i.e., the Lesser Antilles volcanic arc north of Martinique; southern Lesser Antilles, i.e., the Lesser Antilles volcanic arc south of Dominica.

Six analyses (A–F) were conducted using the fossil-calibrated phylogeny resulting from the analysis in BEAST. Models were constructed using dispersal matrices for each time slice in each analysis, incorporating constraints of the hypothesis and landmass availability for each time slice. The fit of each model to the fossil-calibrated phylogeny was reported as a likelihood score.

## Electronic supplementary material

Supplementary Information

## Data Availability

DNA sequences: Genbank accessions FXXXXXX-FXXXXXX (Appendix 1). Phylogenetic data, including alignments and tree files are available at Dryad https://doi.org/XXXX/dryad.XXXXX.
