# Publisher Correction: Island Ancestors and New World Biogeography: A Case Study from the Scorpions (Buthidae: Centruroidinae)

**DOI:** 10.1038/s41598-020-64154-6

**Published:** 2020-04-30

**Authors:** Lauren A. Esposito, Lorenzo Prendini

**Affiliations:** 1 Scorpion Systematics Research Group, Division of invertebrate Zoology, American Museum of natural History, Central Park West at 79th Street, New York, NY 10024-5192 USA; 2 Graduate School and University Center, City University of New York, 365 5th Avenue, New York, NY 10016 USA; 3 Essig Museum of Entomology, 130 Mulford Hall, University of California, Berkeley, CA 94720-3114 USA; 4 Present Address: Institute for Biodiversity Science and Sustainability, California Academy of Sciences, 55 Music Concourse Drive, San Francisco, CA 94118 USA

Correction to: *Scientific Reports* 10.1038/s41598-018-33754-8, published online 05 March 2019

The original version of this Article contained errors.

In Figure 2, representative photos of the major clades of Centruroides: 1) Centruroides scultpuratus (Ewing, 1928), 2) Centruroides hentzi (Banks, 1900), 3) Centruroides gracilis Latreille, 1904, 4) Centruroides rileyi Sissom, 1995 were missing.

Additionally, the Data Availability statement was incorrect.

“DNA sequences: Genbank accessions FXXXXXX-FXXXXXX (Appendix 1). Phylogenetic data, including alignments and tree files are available at Dryad https://doi.org/XXXX/dryad.XXXXX.”

now reads:

“DNA sequences are accessioned in Genbank and listed in Appendix 1.”

Finally, in the Supplementary Information file, Supplementary Figure 1 was omitted.

These errors have been corrected in the HTML and PDF versions of the Article, and in the accompanying Supplementary Information file.

