# Supplementary material for: Island Ancestors and New World Biogeography: A Case Study from the Scorpions (Buthidae: Centruroidinae)
Source: Sci Rep. 2019 Mar 5;9:3500. doi: 10.1038/s41598-018-33754-8 (PMC6401060; doi:10.1038/s41598-018-33754-8)
Supplement: Supplementary file 1 — Supplementary Information [file 41598_2018_33754_MOESM1_ESM.docx]

**Supplementary Information– Island Ancestors and New World Biogeography: A Case Study from the Scorpions (Buthidae: Centruroidinae)**

**Lauren A. Esposito and Lorenzo Prendini**

**Appendix 1**. Terminal taxa, countries and subnational administrative zones of origin, tissue catalog numbers (AMNH), and GenBank accession numbers for 12S rDNA (12S), 16S rDNA (16S), 18S rDNA (18S), 28S rDNA (28S) and Cytochrome *c* Oxidase Subunit I (COI) sequences used in fossil-calibrated phylogenetic analysis of the New World buthid scorpion subfamily Centruroidinae Kraus, 1955.

| **Species** | **Locality** | **Tissue Number** | |  | **Genbank Accession Numbers** | | | |  |
| --- | --- | --- | --- | --- | --- | --- | --- | --- | --- |
|  |  |  | **12S** | | | **16S** | **18S** | **28S** | **COI** |
| *Centruroides arctimanus* Armas, 1976 | Cuba, Holguín Prov. | 7295 | MK478957 | | | MK479008 | MK479059 | MK479110 | MK479161 |
| *Centruroides baergi* Hoffmann, 1932 | Mexico, Oaxaca | 2068 | MK478958 | | | MK479009 | MK479060 | MK479111 | MK479162 |
| *Centruroides balsasensis* Ponce & Francke, 2004 | México, Michoacán | 2010 | MK478959 | | | MK479010 | MK479061 | MK479112 | MK479163 |
| *Centruroides bani* Armas & Marcano Fonduer, 1987 | Dominican Republic, La Altagracia Prov. | 3302 | MK478960 | | | MK479011 | MK479062 | MK479113 | MK479164 |
| *Centruroides barbudensis* Pocock, 1898 | Netherlands Antilles, Saba | 6902 | MK478961 | | | MK479012 | MK479063 | MK479114 | MK479165 |
| *Centruroides bicolor* (Pocock, 1898) | Costa Rica, Puntarenas Prov. | 6112 | MK478962 | | | MK479013 | MK479064 | MK479115 | MK479166 |
| *Centruroides borinquensis* Armas, 1982 | U.S.A., Puerto Rico | 10212 | MK478963 | | | MK479014 | MK479065 | MK479116 | MK479167 |
| *Centruroides chamulaensis* Hoffmann, 1932 | México, Chiapas | 5248 | MK478964 | | | MK479015 | MK479066 | MK479117 | MK479168 |
| C*entruroides edwardsii* (Gervais, 1843) | Ecuador, Loja Prov. | 8384 | MK478965 | | | MK479016 | MK479067 | MK479118 | MK479169 |
| *Centruroides elegans* (Thorell, 1876) | Mexico, Jalisco | 1820 | MK478966 | | | MK479017 | MK479068 | MK479119 | MK479170 |
| *Centruroides exilicauda* (Wood, 1863) | Mexico, Baja California Sur | 1692 | KY981988.1 | | | KY982084.1 | KY981797.1 | KY981893.1 | KY982179.1 |
| *Centruroides exilimanus* Teruel & Stockwell, 2002 | El Salvador, Isla Martín Pérez | 9171 | MK478967 | | | MK479018 | MK479069 | MK479120 | MK479171 |
| *Centruroides farri* (Thorell, 1876) | Jamaica, St. Elizabeth Parish | 5107 | MK478969 | | | MK479020 | MK479071 | MK479122 | MK479173 |
| *Centruroides flavopictus* (Pocock, 1898) | Mexico, Veracruz | 1823 | MK478968 | | | MK479019 | MK479070 | MK479121 | MK479172 |
| *Centruroides fulvipes* (Pocock, 1898) | Mexico, Oaxaca | 8580 | MK478970 | | | MK479021 | MK479072 | MK479123 | MK479174 |
| *Centruroides gracilis* (Latreille, 1804) | Venezuela, Vargas | 1550 | MK478971 | | | MK479022 | MK479073 | MK479124 | MK479175 |
| *Centruroides griseus* (C.L. Koch, 1844) | British Virgin Islands, Guana Is. | 1786 | MK478972 | | | MK479023 | MK479074 | MK479125 | MK479176 |
| *Centruroides guanensis* Franganillo, 1930 | Cuba, Pinar del Rio Prov. | 12563 | MK479007 | | | MK479058 | MK479109 | MK479160 | MK479211 |
| *Centruroides hentzi* (Banks, 1900) | U.S.A., Florida | 1673 | MK478973 | | | MK479024 | MK479075 | MK479126 | MK479177 |
| *Centruroides hoffmanni* Armas, 1996 | Mexico, Chiapas | 5350 | MK478974 | | | MK479025 | MK479076 | MK479127 | MK479178 |
| *Centruroides infamatus* Pocock, 1902 | Mexico, Jalisco | 4709 | MK478975 | | | MK479026 | MK479077 | MK479128 | MK479179 |
| *Centruroides insulanus* (Thorell, 1876) | Jamaica, St. Thomas Parish | 5110 | MK478976 | | | MK479027 | MK479078 | MK479129 | MK479180 |
| *Centruroides insularis* Pocock, 1902 | Mexico, Nayarit | 9528 | MK478977 | | | MK479028 | MK479079 | MK479130 | MK479181 |
| *Centruroides koesteri* Kraepelin, 1912 | Costa Rica, Guanacaste Prov. | 6104 | MK478978 | | | MK479029 | MK479080 | MK479131 | MK479182 |
| *Centruroides limbatus* (Pocock, 1898) | Costa Rica, Puntarenas Prov. | 1957 | MK478979 | | | MK479030 | MK479081 | MK479132 | MK479183 |
| *Centruroides limpidus* (Karsch, 1879) | Mexico, Jalisco | 1911 | MK478980 | | | MK479031 | MK479082 | MK479133 | MK479184 |
| *Centruroides luceorum* Armas, 1999 | U.S.A., Navassa Is. | 9602 | MK478981 | | | MK479032 | MK479083 | MK479134 | MK479185 |
| *Centruroides marcanoi* Armas, 1981 | Dominican Republic, Pedernales Prov. | 10542 | MK478982 | | | MK479033 | MK479084 | MK479135 | MK479186 |
| *Centruroides meisei* Hoffmann, 1938 | Mexico, Guerrero | 10625 | MK478983 | | | MK479034 | MK479085 | MK479136 | MK479187 |
| *Centruroides meridionalis* Hoffmann, 1932 | Mexico, Chiapas | 5252 | MK478984 | | | MK479035 | MK479086 | MK479137 | MK479189 |
| *Centruroides morenoi* Mello-Leitao, 1945 | Jamaica, St. Andrews Parish | 5118 | MK478985 | | | MK479036 | MK479087 | MK479138 | MK479188 |
| *Centruroides nigrescens* (Pocock, 1898) | Mexico, Michoacán | 2005 | MK478986 | | | MK479037 | MK479088 | MK479139 | MK479190 |
| *Centruroides nigrimanus* Pocock, 1898 | Mexico, Oaxaca | 1808 | MK478987 | | | MK479038 | MK479089 | MK479140 | MK479191 |
| *Centruroides nigrovariatus* (Pocock, 1898) | Mexico, Oaxaca | 2033 | MK478988 | | | MK479039 | MK479090 | MK479141 | MK479192 |
| *Centruroides noxius* Hoffmann, 1932 | Mexico, Nayarit | 2071 | MK478989 | | | MK479040 | MK479091 | MK479142 | MK479193 |
| *Centruroides ochraceus* (Pocock, 1898) | Mexico, Quintana Roo | 7666 | MK478990 | | | MK479041 | MK479092 | MK479143 | MK479194 |
| *Centruroides ornatus* Pocock, 1902 | México, Michoacán | 2003 | MK478991 | | | MK479042 | MK479093 | MK479144 | MK479195 |
| *Centruroides platnicki* Armas, 1981 | Turks and Caicos, Caicos | 9051 | MK478992 | | | MK479043 | MK479094 | MK479145 | MK479196 |
| *Centruroides pococki* Sissom & Francke, 1983 | St. Kitts and Nevis, St. Kitts | 9040 | MK478993 | | | MK479044 | MK479095 | MK479146 | MK479197 |
| *Centruroides rileyi* Sissom, 1995 | Mexico, San Luis Potosí | 6445 | KY981992.1 | | | KY982088.1 | KY981801.1 | KY981897.1 | KY982183.1 |
| *Centruroides sasae* Santiago-Blay, 2009 | U.S.A, Puerto Rico, Isla Caja de Muertos | 10239 | MK478994 | | | MK479045 | MK479096 | MK479147 | MK479198 |
| *Centruroides schmidti* Sissom, 1995 | Honduras, Dept. Francisco Morazán | 9172 | KY981993.1 | | | KY982089.1 | KY981802.1 | KY981898.1 | KY982184.1 |
| *Centruroides sculpturatus* Ewing, 1928 | Mexico, Sonora | 2169 | MK478995 | | | MK479046 | MK479097 | MK479148 | MK479199 |
| *Centruroides serrano* Santibanez-Lopez & Ponce-Saavedra, 2009 | Mexico, Oaxaca | 9522 | MK478996 | | | MK479047 | MK479098 | MK479149 | MK479200 |
| *Centruroides simplex* (Thorell, 1876) | Netherlands Antilles, Aruba | 9045 | MK478997 | | | MK479048 | MK479099 | MK479150 | MK479202 |
| *Centruroides sissomi* Armas, 1996 | Mexico, Quintana Roo | 7597 | MK478998 | | | MK479049 | MK479100 | MK479154 | MK479201 |
| *Centruroides suffusus* (Pocock, 1902) | Mexico, Durángo | 5260 | MK478999 | | | MK479050 | MK479101 | MK479151 | MK479203 |
| *Centruroides taino* Armas & Marcano Fondeur, 1987 | Dominican Republic, Pedernales Prov. | 2475 | MK479000 | | | MK479051 | MK479102 | MK479152 | MK479204 |
| *Centruroides tapachulaensis* Hoffmann, 1932 | Mexico, Chiapas | 8578 | MK479001 | | | MK479052 | MK479103 | MK479153 | MK479205 |
| *Centruroides tecomanus* Hoffmann, 1932 | Mexico, Michoacán | 2007 | MK479002 | | | MK479053 | MK479104 | MK479156 | MK479206 |
| *Centruroides testaceus* DeGeer, 1778 | Netherlands Antilles, Bonaire | 9068 | MK479003 | | | MK479054 | MK479105 | MK479155 | MK479207 |
| *Centruroides thorellii* (Kraepelin, 1891) | Guatemala, Sacatepéquez Dept. | 5983 | MK479004 | | | MK479055 | MK479106 | MK479157 | MK479208 |
| *Centruroides tuxtla* Armas, 1996 | Mexico, Chiapas | 3709 | MK479005 | | | MK479056 | MK479107 | MK479158 | MK479209 |
| *Centruroides vittatus* (Say, 1821) | U.S.A., Illinois | 1721 | MK479006 | | | MK479057 | MK479108 | MK479159 | MK479210 |
| *Heteroctenus abudi* (Armas & Marcano Fondeur, 1987) | U.S.A., Puerto Rico, Isla Mona | 10234 | KY981995.1 | | | KY982091.1 | KY981804.1 | KY981900.1 | KY982186.1 |
| *Heteroctenus bonettii* (Armas, 1999) | Dominican Republic, Pedernales Prov. | 2471 | KY981998.1 | | | KY982094.1 | KY981807.1 | KY981903.1 | KY982189.1 |
| *Heteroctenus garridoi* (Armas, 1974) | Cuba, Guantánamo Prov. | 10225 | KY982000.1 | | | KY982096.1 | KY981809.1 | KY981905.1 | KY982191.1 |
| *Heteroctenus junceus* (Herbst, 1800) | Cuba, Guantánamo Prov. | 12613 | KY982001.1 | | | KY982097.1 | KY981810.1 | KY981906.1 | KY982192.1 |
| *Heteroctenus princeps* (Karsch, 1879) | Dominican Republic, La AltagraciaProv. | 12478 | KY982009.1 | | | KY982105.1 | KY981818.1 | KY981914.1 | KY982200.1 |
| *Ischnotelson guanambiensis* (Lenarducci et al., 2005) | Brazil, Bahía | 9669 | KY982014.1 | | | KY982109.1 | KY981823.1 | KY981919.1 | KY982205.1 |
| *Ischnotelson peruassu* Esposito et al., 2017 | Brazil, Minas Gerais | 9937 | KY982017.1 | | | KY982112.1 | KY981826.1 | KY981922.1 | KY982208.1 |
| *Isometrus maculatus* (DeGeer, 1778) | Sri Lanka, Uva Prov. | 1798 | KY982016.1 | | | KY982111.1 | KY981825.1 | KY981921.1 | KY982207.1 |
| *Jaguajir agamemnon* (C.L. Koch, 1839) | Brazil, Maranhão | 9692 | KY982018.1 | | | KY982113.1 | KY981827.1 | KY981923.1 | KY982209.1 |
| *Jaguajir pintoi* (Mello-Leitão, 1932) | Guyana, Upper Takutu-Upper Essequibo Region | 8278 | KY982029.1 | | | KY982124.1 | KY981838.1 | KY981934.1 | KY982220.1 |
| *Jaguajir rochae* (Borelli, 1910) | Brazil, Paraiba | 1775 | KY982033.1 | | | KY982128.1 | KY981842.1 | KY981938.1 | KY982224.1 |
| *Physoctonus debilis* (C.L. Koch, 1840) | Brazil, Piauí | 9678 | KY982048.1 | | | KY982143.1 | KY981857.1 | KY981952.1 | KY982238.1 |
| *Physoctonus striatus* Esposito et al., 2017 | Brazil, Bahía | 9681 | KY982051.1 | | | KY982146.1 | KY981860.1 | KY981955.1 | KY982241.1 |
| *Rhopalurus caribensis* Teruel & Roncallo, 2008 | Colombia, Magdalena Dept. | 13167 | KY982052.1 | | | KY982147.1 | KY981861.1 | KY981956.1 | KY982242.1 |
| *Rhopalurus laticauda* Thorell, 1876 | Venezuela, Aragua | 10046 | KY982054.1 | | | KY982149.1 | KY981863.1 | KY981958.1 | KY982244.1 |
| *Rhopalurus ochoai* Esposito et al., 2017 | Venezuela, Edo. Trujillo | 5504 | MF508621.1 | | | MF508628.1 | **–** | MF402014.1 | MF508635.1 |
| *Tityus atriventer* Pocock, 1897 | Grenada, St. Andrew Parish | 9033 | KY982074.1 | | | KY982169.1 | KY981883.1 | KY981978.1 | KY982264.1 |
| *Tityus metuendus* Pocock, 1897 | Guyana, Upper Takutu-Upper Essequibo Region | 1546 | KY982079.1 | | | KY982174.1 | KY981888.1 | KY981983.1 | KY982269.1 |
| *Troglorhopalurus lacrau* (Lourenço & Pinto-da-Rocha 1997) | Brazil, Bahía | 10211 | MF508625.1 | | | MF508632.1 | MF508615.1 | MF508618.1 | MF508639.1 |
| *Troglorhopalurus translucidus* Lourenço et al., 2004 | Brazil, Bahía | 9668 | MF508627.1 | | | MF508634.4 | MF508617.1 | MF508620.1 | MF508641.1 |
